# *Trichinella spiralis* Paramyosin Induces Colonic Regulatory T Cells to Mitigate Inflammatory Bowel Disease

**DOI:** 10.3389/fcell.2021.695015

**Published:** 2021-07-15

**Authors:** Chunyue Hao, Wei Wang, Bin Zhan, Zixia Wang, Jingjing Huang, Ximeng Sun, Xinping Zhu

**Affiliations:** ^1^Department of Medical Microbiology and Parasitology, School of Basic Medical Sciences, Capital Medical University, Beijing, China; ^2^Department of Pediatrics, National School of Tropical Medicine, Baylor College of Medicine, Houston, TX, United States

**Keywords:** r*Ts*Pmy, *Trichinella spiralis*, regulatory T cells, inflammatory bowel disease, immunomodulation

## Abstract

Helminth infection modulates host regulatory immune responses to maintain immune homeostasis. Our previous study identified *Trichinella spiralis* paramyosin (*Ts*Pmy) as a major immunomodulatory protein with the ability to induce regulatory T cells (Tregs). However, whether *Ts*Pmy regulates gut Tregs and contributes to intestinal immune homeostasis remains unclear. Here we investigated the therapeutic effect of recombinant *Ts*Pmy protein (r*Ts*Pmy) on experimental colitis in mice, and elucidated the roles and mechanisms of colonic Tregs induced by r*Ts*Pmy in ameliorating colitis. Acute colitis was induced by dextran sodium sulfate (DSS) in C57BL/6J mice, and chronic colitis was induced by naïve T cells in Rag1 KO mice. Mice with colitis were pre-treated with r*Ts*Pmy intraperitoneally, and clinical manifestations and colonic inflammation were evaluated. Colonic lamina propria (cLP) Tregs phenotypes and functions in DSS-induced colitis were analyzed by flow cytometry. Adoptive transfer of cLP Tregs treated by r*Ts*Pmy into Rag1 KO chronic colitis was utilized to verify Tregs suppressive function. r*Ts*Pmy ameliorated the disease progress of DSS-induced colitis, reduced pro-inflammatory responses but enhanced regulatory cytokines production in DSS-induced colitis. Moreover, r*Ts*Pmy specifically stimulated the expansion of thymic-derived Tregs (tTregs) rather than the peripherally derived Tregs (pTregs) in the inflamed colon, enhanced the differentiation of effector Tregs (eTregs) with higher suppressive function and stability in colitis. This study describes the mechanisms of colonic Tregs induced by the *Trichinella*-derived protein r*Ts*Pmy in maintaining gut immune homeostasis during inflammation. These findings provide further insight into the immunological mechanisms involved in the therapeutic effect of helminth-derived proteins in inflammatory bowel diseases.

## Introduction

Inflammatory bowel disease (IBD) is widely described as an autoimmune disease characterized by chronic recurrent inflammation in the gastrointestinal tract as a result of excessive immune responses to gut lumen antigens in genetically susceptible individuals. The two most common IBDs are Crohn’s disease (CD) and ulcerative colitis (UC) (Xavier and Podolsky, 2007; Maloy and Powrie, 2011). Abdominal pain, diarrhea, and weight loss are the most common symptoms in patients with IBD. The incidence and prevalence of IBD are rising worldwide, particularly in newly industrialized regions (Kaplan, 2015; Zuo et al., 2018). Despite an increasing repertoire of therapeutic targets focusing on lymphocyte trafficking and activation, gut barrier function and matrix remodeling, and even manipulation of gut microbiota (Neurath, 2017), a large number of patients still have chronic intestinal inflammation.

Foxp3^+^ regulatory T cells (Tregs) represent a unique CD4^+^ T-cell subset essential for maintaining immune balance and homeostasis (Ohkura et al., 2013). The gut, especially the colon, has highly abundant Tregs in the lamina propria (LP), accounting for about 20–30% of total CD4^+^ T cells, and is responsible for adapting to resident antigens in the intestine such as commensal bacteria and food antigens (Tanoue et al., 2016). Tregs restrict inflammatory responses and maintain immune tolerance via various molecular mechanisms at a multitude of cellular levels, including (i) inhibition of T-cell proliferation and effector functions through the anti-inflammatory cytokines IL-10, TGF-β, and IL-35, (ii) apoptosis of effector T cells (Teffs) induced by starvation of IL-2, (iii) metabolic disruption of Teffs by converting ATP into adenosine, (iv) direct cytolysis against Teffs by galectin-1, granzyme B and perforin, and (v) contact-dependent neutralization of dendritic cell (DC) function by the interaction of Treg-derived CTLA4/TIGIT and CD80/CD155 on DCs (Shevach, 2009, 2018).

There is a shred of definitive evidence that patients with IBD have defects in quantity and quality of Tregs (Abdel-Motal et al., 2019), coupled with the fact that the colon is a primary site of inflammation; therefore, enhancing colonic Treg activity has become a compelling strategy for the immunotherapy of IBD. Several studies demonstrated that it was feasible to expand polyclonal Tregs from patients *in vitro* under good manufacturing practice (GMP) condition as a source of therapy, in which these Tregs sustained their stability and suppression capabilities (Canavan et al., 2016; Mathew et al., 2018). Antigen-specific Tregs are believed to be more effective than polyclonal Tregs (Clough et al., 2020); however, no antigen has yet been identified as a definite cause in IBD. Nonetheless, a French study reported a clinical trial with ovalbumin (OVA)-specific Tregs showing dose-related therapeutic efficacy in refractory CD (Desreumaux et al., 2012). More clinical trials using autologous Tregs expanded *in vitro* are ongoing for immunotherapy of CD (ClinicalTrails.gov Identifier: NCT03185000).

The increase in the prevalence of IBD has been attributed to improved hygiene and the disappearance of intestinal helminth and other infections. Based on the “hygiene hypothesis,” a lack of early childhood exposure to multiple antigens increases susceptibility to allergies and autoimmune diseases including IBD. It is possible that intestinal immune homeostasis requires the presence of helminth infections (Gause and Maizels, 2016; Bach, 2018). The differentiation, maintenance, and functional maturation of gut Tregs are orchestrated via various specific signals from the lumen environment (Tanoue et al., 2016). The activation of Tregs has emerged as a central explanation for the beneficial role of helminth infections in moderating inflammatory diseases such as allergies and autoimmune disorders including IBD (Weinstock, 2015; Gazzinelli-Guimaraes and Nutman, 2018). There is growing evidence demonstrating that helminths resident in the gut continuously release immunoregulatory small molecules to modulate intestinal Tregs to benefit immune homeostasis locally or systemically (Reynolds et al., 2015; Gause and Maizels, 2016; Lopes et al., 2016). Natural infection with intestinal helminths reduced pathology in patients with UC (Buning et al., 2008; Broadhurst et al., 2010). Clinical trials with eggs of *Trichuris suum* and hookworm *Necator americanus* decreased symptoms in patients with chronic CD or UC (Cheifetz et al., 2017). Although the benefits of helminth infection in colitis have been extensively investigated, the mechanism related to effect of helminth-expanded colonic Tregs on the mitigation of IBD has not been studied extensively (Lopes et al., 2016), even though several studies have showed that infection with intestinal helminths stimulated Tregs in the colon (Taylor et al., 2012) and were associated with therapeutic effects in colitis (Hang et al., 2013). Because of safety concerns in helminth therapy, it is more feasible and practical to identify the specific helminth-derived molecules that stimulate Tregs’ response as an immunotherapeutic target for chronic IBD and other inflammatory diseases.

*Trichinella spiralis* is a tissue-dwelling nematode with adult worms residing in small intestinal mucosa and larvae migrating to striated muscle to form encapsulated larvae. In order to survive in a hostile environment, *T. spiralis* adult worms and larvae secrete various proteins or other small molecules to modulate the host’s immune system to counter the host’s immunological attack (Qi et al., 2018; Song et al., 2018). Our previous study found that excretory/secretory (ES) products of *T. spiralis* adult worms are able to induce Treg proliferation through activating dendritic cells (Sun et al., 2019). Experimental treatment with *T. spiralis* ES products significantly reduced colitis induced by dextran sodium sulfate (DSS) in a mouse model through stimulating Tregs (Yang et al., 2014; Wang et al., 2020) or macrophage M2 polarization (Wang et al., 2020) to reduce inflammation in the colon. The adult worm extracts of *T. spiralis* also stimulated regulatory cytokines and reduced OVA-induced airway inflammation (Yuan et al., 2019). In an effort to identify the effective components in the nematode-secreted proteins that play immunomodulatory functions in host immune systems, it was found that paramyosin as a structural protein expressed on the surface and secreted products of *T. spiralis* larval and adult worms (*Ts*Pmy) is one of the major proteins involved in the immunomodulation of the host immune response through binding to human complement C1q (Sun et al., 2015; Wang et al., 2018) and C8/C9 (Zhang et al., 2011; Zhao et al., 2014) to inhibit complement response. The C9 binding domain of r*Ts*Pmy coupled with a membrane-bound signal reduced complement-related arthritis in a mouse model (Chen et al., 2020). Also, r*Ts*Pmy was able to activate systemic Tregs and induce the differentiation of Tregs through tolerogenic DCs (Guo et al., 2016). However, there is little knowledge about whether r*Ts*Pmy modulates intestinal Tregs and how these Tregs induced by r*Ts*Pmy maintains intestinal immune homeostasis in the inflammatory environment. In the present study, we demonstrated for the first time that r*Ts*Pmy promotes the expansion of thymic-derived Treg cells (tTregs), and maintained the stability and suppressive function of Tregs in the colon as the major mechanism involved in the therapeutic effect of r*Ts*Pmy in experimental colitis in mice.

## Materials and Methods

### Animals

Female C57BL/6J mice (6–8 weeks) were provided by Beijing Vital River Laboratory Animal Technology Co., Ltd (Beijing, China). Female Foxp3^*eGFP*^ reporter mice (B6.Cg-Foxp3^TM 2*Tch*^/J) and Rag1 KO mice (B6.129S7-Rag1^TM 1*Mom*^/J) were purchased from Jackson Laboratory (Bar Harbor, ME, United States). All mice were housed under specific pathogen-free conditions in the Laboratory Animal Services Center of Capital Medical University (Beijing, China) according to the NIH Guidelines for the Care and Use of Laboratory Animals. All experimental procedures were approved by the Capital Medical University Animal Care and Use Committee under projects AEEI-2017-140, AEEI-2016-008 and AEEI-2017-133.

### Recombinant Protein Preparation

*Trichinella spiralis* paramyosin (GenBank# ABO09862.1) was expressed as recombinant protein (r*Ts*Pmy) in the baculovirus insect expression system (Invitrogen, Carlsbad, CA, United States). The r*Ts*Pmy with hexahistidine-tag at the C-terminus was expressed as partially soluble protein. After being denatured with guanidine hydrochloride, r*Ts*Pmy was purified by Ni-affinity chromatography (GE Healthcare, Boston, MA, United States) followed by a refolding process using a Protein Refolding Kit (Novagen/Merck KGaA, Darmstadt, Germany). The refolded soluble r*Ts*Pmy was characterized by SDS-PAGE and could be recognized by anti-r*Ts*Pmy sera by western blot.

### DSS Induced-Colitis and Pathology

Acute colitis was induced in female C57BL/6J mice with DSS as previously described (Wirtz et al., 2017). In brief, mice received 3% DSS (36,000–50,000 MW, MP Biomedicals, Solon, OH, United States) in drinking water for 6 days followed by regular drinking water for 2 days. The pathological changes of DSS-induced colitis were evaluated by the following criteria:

#### Clinical Scoring

The clinical scoring of colitis was measured by the weight loss, stool consistency (diarrhea), and rectal bleeding as described (Yang et al., 2014; Table 1) from day 0 to day 8 using following criteria. The disease activity index (DAI) was calculated based on the sum of these scoring criteria.

**TABLE 1:** The criteria for scoring DSS-induced colitis.

Score	0	1	2	3	4
Weight loss	None	1–5%	5–10%	10–15%	>15%
Stool consistency	Well-formed pellets	Between	Pasty and semi-formed stools	Between	Liquid stools
Rectal bleeding	None	Between	Slight bleeding	Between	Gross bleeding

#### Histological Scoring

On day 8, all mice were euthanized, and the distal colons were fixed in 4% paraformaldehyde. Paraffin-embedded sections (5 μm) were stained with hematoxylin and eosin (H&E). Histological activity score was assessed as the sum of two parameters as follows (Okayasu et al., 1990): Crypt damage, 0 = none; 1 = basal 1/3 lost; 2 = basal 2/3 lost; 3 = crypts lost but surface epithelium present; 4 = entire crypt and epithelium lost. Inflammatory cell infiltration, 0 = none; 1 = infiltration around crypt bases; 2 = infiltration in the muscularis mucosa; 3 = extensive infiltration in the muscularis mucosa with edema; 4 = infiltration of the submucosa.

### Myeloperoxidase (MPO) Staining

The colon tissue sections were reacted with rat anti-MPO mAb (Servicebio, Wuhan, China) at 1:500 dilution at 4°C overnight and then incubated with HRP-conjugated rabbit anti-rat IgG at room temperature for 50 min, followed by the diaminobenzidin (DAB) chromogenic reaction for MPO detection. The nuclei on all slides were counterstained with hematoxylin.

### Treatment of r*Ts*Pmy in DSS-Induced Colitis

Female C57BL/6J mice were intraperitoneally treated with 20 μg of r*Ts*Pmy for three times at 2-week intervals. Right after the last treatment, all mice were challenged with DSS daily as described above. Each mouse was boosted with another treatment of r*Ts*Pmy (20 μg) on the day 5 of DSS induction. The pathological changes of DSS-induced colitis were measured as above.

### Immunostaining of CD4^+^ T Cells in Colon Sections

To identify CD4^+^ T cells, colon sections were obtained from Foxp3^*eGFP*^ reporter mice and reacted with rabbit anti-CD4 mAb (Servicebio, Wuhan, China) at 1:400 dilution at 4°C overnight and then incubated with Cy3-conjugated goat anti-rabbit IgG secondary antibody for CD4 detection. All slides were counterstained for nuclei with DAPI (1:5000 dilution, Sigma, St. Louis, MO, United States). Slides were observed using the Panoramic 250 Flash system (3DHISTECH, Budapest, Hungary) with Panoramic Viewer software (3DHISTECH, Budapest, Hungary).

### Isolation of Lamina Propria Cells

The colons were removed and placed in Ca^2+^, Mg^2+^-free Hank’s balanced salt solution (HBSS; Gibco, Carlsbad, CA, United States). Colonic lamina propria (cLP) cells were isolated using the Lamina Propria Dissociation Kit (Miltenyi Biotec, GmBH, Cologne, Germany) in a gentleMACS Octo Dissociator at 37°C. In brief, the colons were incubated with Ca^2+^/Mg^2+^-free HBSS containing 1 mM dithiothreitol (DTT, Absin, Shanghai, China), 5 mM EDTA (Calbiochem, Merck, Darmstadt, Germany), 5% FBS (Gibco, Carlsbad, CA, United States), 100 U/mL penicillin, 100 U/mL streptomycin and 10 mM HEPES (Absin, Shanghai, China) for 50 min for two times at 37°C on a MACSmix Tube Rotator to remove epithelial layers. The remaining colon pieces were digested with an enzyme mix from a Lamina Propria Dissociation Kit for 30 min. The suspension was filtered through a MACS SmartStrainer (40 μm) and the single-cell suspension was washed once with PBS containing 5% FBS.

### Cytokine Assay

Epithelial layer cells were cultured with 25 ng/ml PMA (phorbol 12-myristate-13-acetate) and 1 μg/ml ionomycin (Sigma) for 48 h. The cLP cells were stimulated with 2 μg/mL anti-CD3 and 1 μg/mL anti-CD28 mAbs (Biolegend, San Diego, CA, United States) for 48 h. Different cytokines in cell culture supernatants were measured by the multiplex Luminex immunoassay platform or ELISA.

### Flow Cytometry

All detected antibodies are listed in Table 2. The suspended cells were pre-incubated with Zombie Violet Fixable Viability Kit (Biolegend, San Diego, CA, United States) to detect dead cells. FcγR was blocked with anti-CD16/CD32 antibody (clone 93, eBioscience, San Diego, CA, United States). For intracellular cytokine staining, LP cells (2 × 10^6^/mL) were stimulated with a Cell Activation Cocktail (with Brefeldin A) (Biolegend) in complete medium (RPMI 1640 containing 10% FBS, 100 U/mL penicillin, 100 μg/mL streptomycin) in 24-well plates for 5 h, then stained for intracellular cytokines IL-17A and IFN-γ using a Cytofix/Cytoperm kit (BD Bioscience, Franklin Lakes, NJ, United States) after staining surface antigens CD3ε and CD4. For intranuclear transcription factors staining, LP cells were stained for surface antigens CD3ε, CD4, and CD25, followed by staining intranuclear transcription factors for Foxp3, GATA3, and Helios using the Foxp3/Transcription Factor Fixation/Permeabilization staining buffer kit (eBioscience).

**TABLE 2:** The key resources in this study.

Reagent or resource	Source	Identifier
**Antibodies**		
Anti- CD16/CD32	eBioscience (United States)	Cat.# 14-0161-85, RRID:AB_467134
Anti-CD3	eBioscience	Cat.# 46-0032-80; RRID:AB_1834428
Anti-CD4	eBioscience	Cat.# 12-0041-82; RRID:AB_465506
Anti-CD4	eBioscience	Cat.# 17-0042-82; RRID:AB_469323
Anti-CD4	eBioscience	Cat.# A15384; RRID:AB_2534398
Anti-CD25	eBioscience	Cat.# 17-0251-82; RRID:AB_469366
Anti-Foxp3	eBioscience	Cat.# A18662; RRID:AB_2535451
Anti-Helios	eBioscience	Cat.# 25-9883-42; RRID:AB_2637136
Anti-CD45RB	Biolegend (United States)	Cat.# 103308; RRID:AB_313015
Anti-IFN-γ	eBioscience	Cat.# 25-7311-82; RRID:AB_469680
Anti-IL-17A	eBioscience	Cat.# 25-7177-82; RRID:AB_10732356
Anti-GATA3	eBioscience	Cat.# 12-9966-42; RRID:AB_1963600
Anti-TIGIT	Biolegend	Cat.# 142109; RRID:AB_2566572
Anti-CTLA4	Tonbo Biosciences (United States)	Cat.# 50-1522, RRID:AB_262178
Anti-CD62L	eBioscience	Cat.# 25-0621-82; RRID:AB_469633
Anti-CD44	eBioscience	Cat.# 48-0441-82; RRID:AB_1272246
Anti-CD4	Abcam (United Kingdom)	Cat.# ab183685, RRID:AB_2686917
Cy3 conjugated Goat Anti-Rabbit IgG (H + L)	Servicebio (China)	Cat.# GB21303, RRID:AB_2861435
Mounting Medium With DAPI	Abcam (United Kingdom)	Cat.# ab104139
Anti-myeloperoxidase (MPO)	Servicebio (China)	Cat.# GB11224, RRID:AB_2814688
**Chemicals, critical commercial assays, recombinant proteins**		
Dextran Sulfate Sodium Salt, DSS	MP Biomedicals (United States)	Cat.# 160110
ProcartaPlex 7 Plex Kit	eBioscience	Cat.# PPX-07
IL-17A ELISA Kit	Invitrogen (United States)	Cat.# 88-7371-88, RRID:AB_2575104
IL-17F ELISA Kit	Invitrogen	Cat.# 88-7472-88, RRID:AB_2575125
IL-6 ELISA Kit	Invitrogen	Cat.# 88-7064-88, RRID:AB_2574990
IFN-γ ELISA Kit	Invitrogen	Cat.# 88-7314-88, RRID:AB_2575070
IL-10 ELISA development Kit	Mabtech (Sweden)	Cat.# 3432-1H-6
IL-10 ELISA Kit	Invitrogen	Cat.# 88-7105-88, RRID:AB_2574997
IL-4 ELISA Kit	Invitrogen	Cat.# 88-7044-88, RRID:AB_2574970
IL-5 ELISA Kit	Invitrogen	Cat.# 88-7054-88, RRID:AB_2574980
IL-13 ELISA Kit	Invitrogen	Cat.# 88-7137-88, RRID:AB_2575026
Lamina Propria Dissociation Kit	Miltenyi Biotec (Germany)	Cat.# 130-097-410
Cell Activation Cocktail (with Brefeldin A)	Biolegend	Cat.# 423304
Zombie Violet^TM^ Fixable Viability Kit	Biolegend	Cat.# 423114
BD Cytofix/Cytoperm^TM^ Fixation/Permeabilization Kit	BD Biosciences (United States)	Cat.# 554714, RRID:AB_2869008
Foxp3/Transcription Factor Staining Buffer Set	eBioscience	Cat.# 00-5523-00

The stained samples were detected on BD LSRFortessa with DIVA software (BD Biosciences) or Cytek Aurora Spectral Flow Cytometry with SpectroFlo2.2.0 (Cytek Biosciences, San Jose, CA, United States). For Treg subsets, the lymphocytes were gated by FSC vs. SSC, and the singlets were gated by FSC-A and FSC-H, and CD3^+^CD4^+^ T cells were gated by CD3 and CD4 fluorescencent antibodies, Foxp3^+^ Treg cells were gated by Foxp3 and CD4 fluorescencent antibodies. For Th17, Th1, and Treg cells, the lymphocytes were gated by FSC vs. SSC, and the singlets were gated by FSC-A and FSC-H, and CD3^+^CD4^+^ T cells were gated by CD3 and CD4 fluorescencent antibodies. In each experiment, the collected cells were pooled and stained with one specific directly labeled antibody to set up the compensation parameters. Background fluorescence was assessed by staining with isotype-matched control mAbs. Data were analyzed using FlowJo software (Tomy Digital Biology, Tokyo, Japan).

### Adoptive Transfer of Naïve T Cells and Treg Cells to Rag1 KO Mice

To establish T-cell-induced colitis in Rag1 KO mice lacking mature T and B lymphocytes, CD4^+^ T cells were isolated from spleens of Foxp3^*eGFP*^ reporter mice using the MACS CD4^+^ T-cell Isolation Kit (Miltenyi Biotec). CD45RB^*hi*^CD4^+^Foxp3^–^ naïve T cells were purified via FACS sorting on FACSAria IIIu (BD Biosciences) to reach over 98%. The naïve T cells were adoptively transferred into Rag1 KO mice (1 × 10^6^/0.1 mL per mouse) through tail vein injection. To observe the role of r*Ts*Pmy-induced Tregs in reducing naïve T cell-induced colitis in Rag1 KO mice, 1 week after being transferred with naïve T cells, the Rag1 KO mice were passively transferred with 1 × 10^5^ Treg cells isolated from cLP of Foxp3^*eGFP*^ reporter mice treated with 20 μg r*Ts*Pmy or PBS for four times. The body weight was recorded weekly. At 4 weeks after Treg cell transfer, the severity of colitis was evaluated by H&E staining and ELISA.

### Statistical Analysis

Data are expressed as the mean ± SEM, and the differences between groups were analyzed using either unpaired two-tailed Student’s *t*-test or one-way ANOVA with the Bonferroni correction. A value of *p* < 0.05 is considered statistically significant.

## Results

### *T. spiralis* Recombinant Paramyosin Ameliorates DSS-Induced Acute Colitis

**FIGURE 1:**
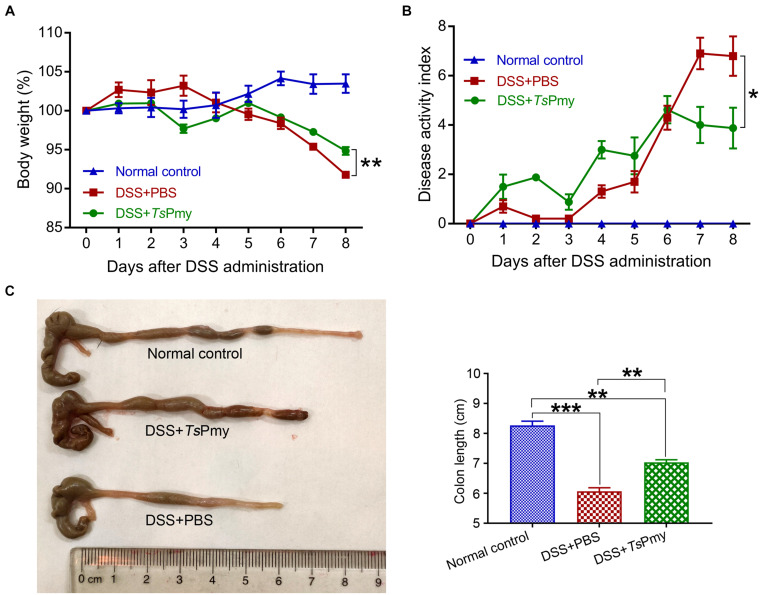
Treatment with r*Ts*Pmy alleviates clinical manifestations and colon shortening in DSS-induced acute colitis. Each mouse was treated with 20 μg of r*Ts*Pmy three times before being challenged with 3% DSS for 6 days. **(A)** Body weight changes (relative to initial weight) (*n* = 5). **(B)** The whole diseases activity index changes (*n* = 5). **(C)** Representative colons from each group are shown on the left; the average colon length from each group is shown on the right (*n* = 5). The data represent the mean ± SEM. *p < 0.05, **p < 0.01, ***p < 0.001.

**FIGURE 2:**
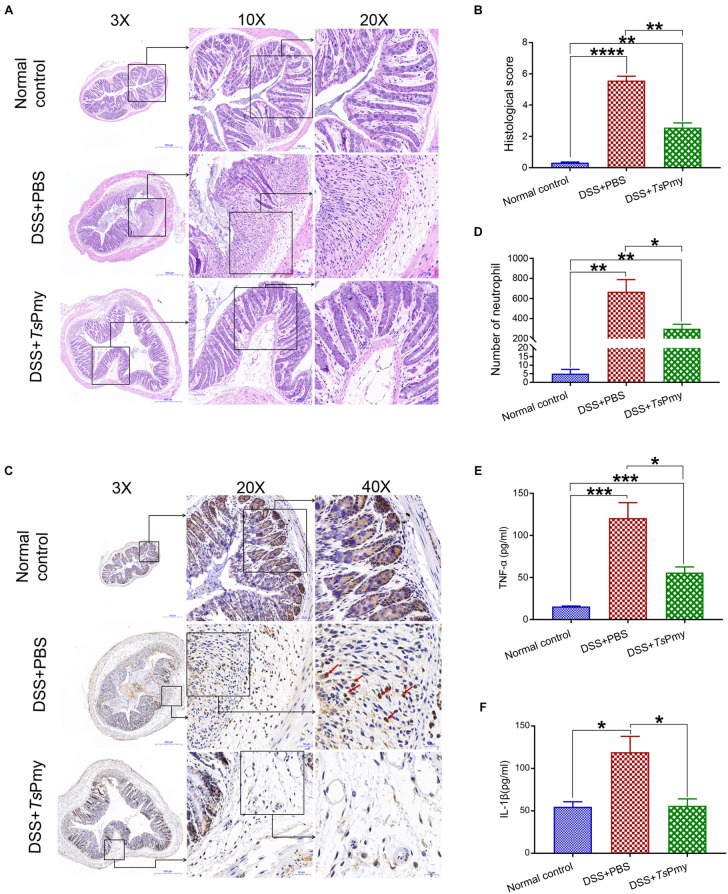
Treatment with r*Ts*Pmy improves the histopathological changes of DSS-induced acute colitis. **(A)** The representative histopathological sections of distal colon at objective 3x, 10x, and 20x. **(B)** The histological scores of colon in each group (*n* = 5). **(C)** Immunohistochemical (IHC) staining of myeloperoxidase (MPO) reflecting neutrophil infiltration (red arrows) in the distal colon. **(D)** The number of neutrophils of whole cross-section of distal colon in each group (*n* = 5). **(E)** The level of cytokines TNF-α derived by colonic epithelial layers cells (*n* = 5). **(F)** The level of cytokines IL-1β of colon homogenate (*n* = 5). The data represent the mean ± SEM. *p < 0.05, **p < 0.01, ***p < 0.001, ****p < 0.0001.

To evaluate whether r*Ts*Pmy has prophylactic and therapeutic effects on DSS-induced acute colitis, mice were each intraperitoneally administered 20 μg r*Ts*Pmy for three times before DSS induction and boosted with one more treatment 5 days after DSS induction. The DSS-induced mice without treatment had typical clinical colitis, including progressive weight loss and diarrhea with bloody feces from day 3 to day 8 after DSS induction. In contrast, treatment with r*Ts*Pmy significantly reduced these clinical manifestations and the disease activity index as well compared to DSS-induced mice without treatment (receiving PBS only) (Figures 1A,B). In terms of pathological changes, the colons in mice with DSS-induced colitis were significantly shortened due to severe inflammatory edema, whereas, treatment with r*Ts*Pmy inhibited the colon shortening (Figure 1C). Histopathological sections displayed severe loss of crypts and infiltration of inflammatory cells (Figures 2A,B), especially neutrophils within the cLP extending to the muscularis mucosae and submucosa (Figures 2C,D), whereas administration of r*Ts*Pmy significantly alleviated the pathologic damage and reduced infiltration of inflammatory cells, including neutrophils, in the colon (Figures 2A–D). The colitis-characterized pro-inflammatory cytokines such as TNF-α secreted by epithelial layer cells and IL-1β of colon homogenate were significantly decreased in r*Ts*Pmy-treated mice in comparison to PBS-treated mice (Figures 2E,F). These observations indicate that r*Ts*Pmy suppresses the development of acute DSS-induced colitis and reduces colon inflammation.

### r*Ts*Pmy Reduces Inflammatory Responses and Enhances Regulatory Cytokines in cLP of Mice With DSS-Induced Colitis

**FIGURE 3:**
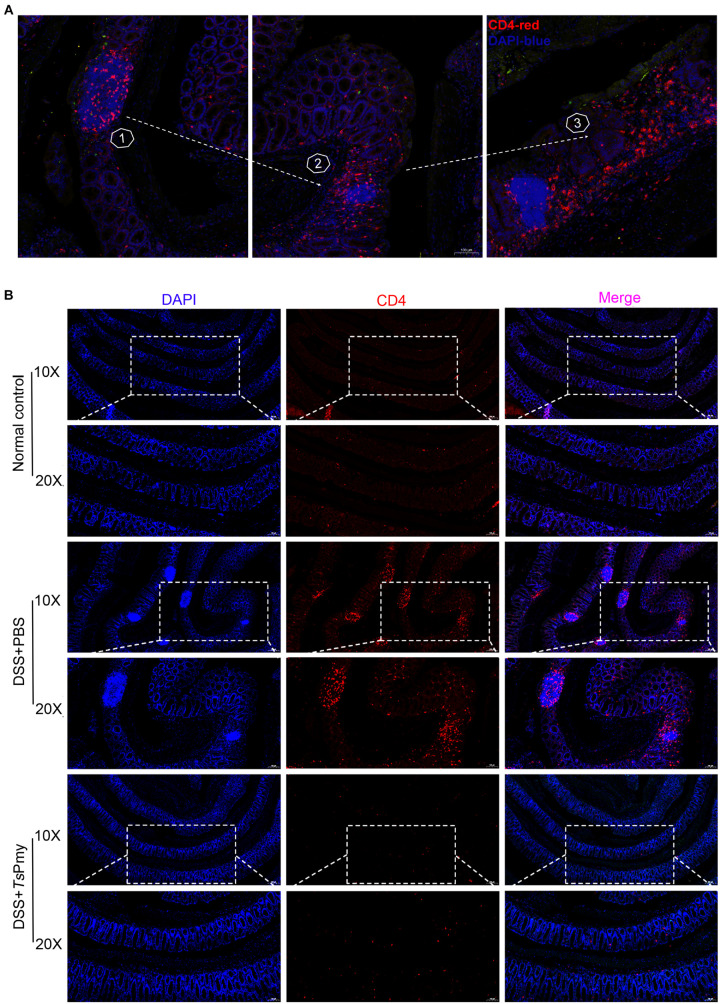
Treatment with r*Ts*Pmy reduces the recruitment of CD4^+^ effector cells in the isolated lymphoid follicles of mice with DSS-induced colitis. Immunofluorescent staining of the distal colon sections with antibodies for CD4 (red), counterstained with DAPI (blue). **(A)** Pathological process of DSS-induced colitis with increased recruitment of CD4^+^ effector cells in ILFs. Foxp3^*eGFP*^ mice were used. **(B)** Distal colon sections of mice from different groups on day 5. Illustrations are representative of 3 mice per group. ILFs, isolated lymphoid follicles.

**FIGURE 4:**
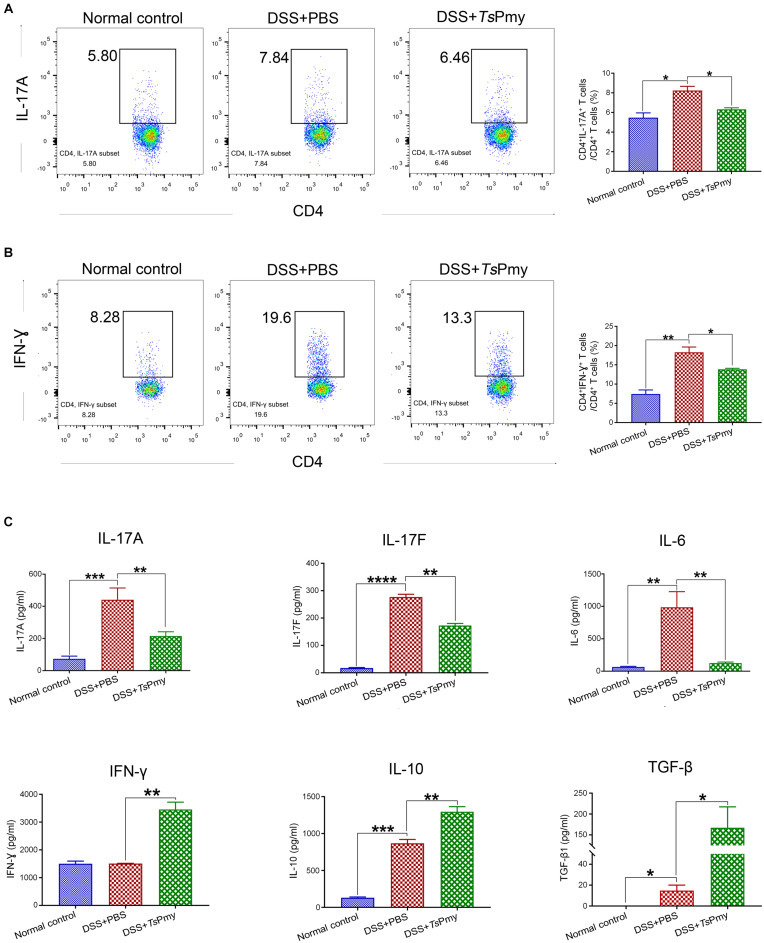
Treatment with r*Ts*Pmy reduces pro-inflammatory responses and enhances regulatory cytokines in cLP of mice with DSS-induced colitis. Lamina propria mononuclear cells (LPMCs) were isolated from distal colons of mice treated with r*Ts*Pmy or PBS on day 8. Flow cytometry shows CD3^+^CD4^+^ expressed with IL-17A **(A)** or IFN-γ **(B)**. The gating strategy is shown in Supplementary Figure 1. The statistical analyses of the percentage of cells are shown on the right (*n* = 5). **(C)** After being stimulated with anti-CD3/CD28 mAbs for 48 h, LPMCs secretions of IL-17A, IL-17F, IL-6, IFN-γ, IL-10, and TGF-β were measured in the culture supernatants by Luminex or ELISA. The data represent the mean ± SEM (*n* = 3–5). *p < 0.05, **p < 0.01, ***p < 0.001, ****p < 0.0001. cLP, colonic lamina propria.

Excessive and prolonged activation of CD4^+^ effector T cells is the direct cause of intestinal inflammation and tissue damage in patients with IBD (Caruso et al., 2020). In this study, we observed that CD4^+^ T cells were significantly recruited to the isolated lymphoid follicles (ILFs) in the colon of mice with DSS-induced colitis (Figure 3A), indicating CD4^+^ effector T cells are involved in the inflammatory process of colitis. Treatment with r*Ts*Pmy significantly reduced the infiltration of CD4^+^ effector T cells in the colon of DSS-treated mice (Figure 3B). After being treated with r*Ts*Pmy, the CD3^+^CD4^+^ T cells expressing IL-17A and IFN-γ were significantly reduced in the colonic lamina propria mononuclear cells (LPMCs) of mice with DSS-induced colitis compared to group treated with PBS only (Figures 4A,B), indicating the colitis-related inflammatory Th1 and Th17 responses were inhibited by *Ts*Pmy treatment.

The pro-inflammatory cytokine IL-17A, IL-17F, and IL-6 derived by LPMCs were significantly reduced in r*Ts*Pmy-treated mice; however, the level of IFN-γ was unexpectedly increased (Figure 4C). Meanwhile, the regulatory cytokines IL-10 and TGF-β were significantly increased in LPMCs of mice treated with r*Ts*Pmy compared to group that received PBS only (Figure 4C).

### r*Ts*Pmy Promotes Thymus-Derived Tregs but Not Peripherally Derived Tregs in cLP of Mice With Colitis

**FIGURE 5:**
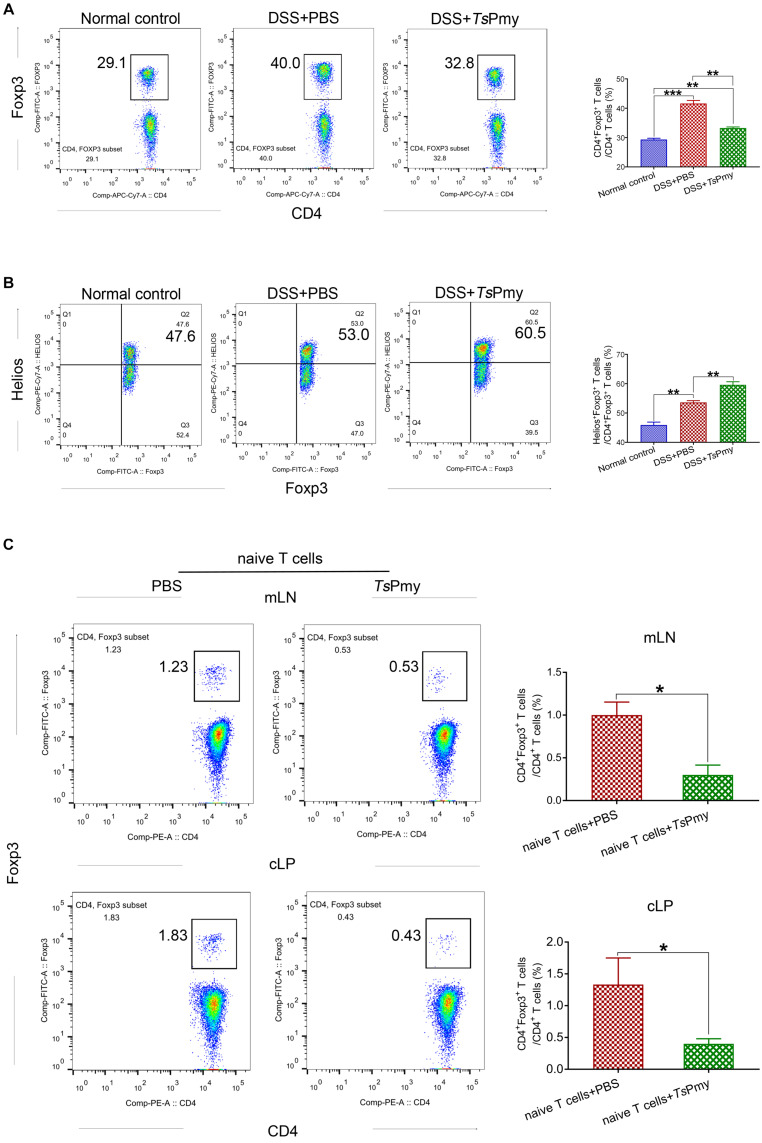
Treatment with r*Ts*Pmy specifically increases Helios^+^ tTreg expansion but could not induce pTreg differentiation in mLN and cLP in colitis. **(A)** The percentage of CD4^+^Foxp3^+^ Tregs in cLP from r*Ts*Pmy- or PBS-treated mice. The gating strategy is shown in Supplementary Figure 2. Statistical analysis is shown on the right (*n* = 5). **(B)** The frequency of Helios^+^ cells in Foxp3^+^ T cells of cLP from r*Ts*Pmy- or PBS-treated mice. The gate strategy is shown in Supplementary Figure 3. Statistical analysis is shown on the right (*n* = 5). **(C)** The percentage of Foxp3^+^ (eGFP) cells in CD3^+^CD4^+^ T cells of mLN (top) and cLP (bottom) at 5 weeks after CD4^+^Foxp3^–^CD45RB^*hi*^ naïve T-cell adoptive transfer into Rag1 KO mice. The gate strategy is shown in Supplementary Figure 4. Frequencies are shown on the right (*n* = 3). Naïve T cells were from Foxp3^*eGFP*^ mice. Results are shown as mean ± SEM. *p < 0.05, **p < 0.01, ***p < 0.001. mLN, mesenteric lymph nodes. cLP, colonic lamina propria. See also in Supplementary Figure 5.

CD4^+^Foxp3^+^ Tregs are most abundant in the colonic mucosa and regulate the immune system in the intestine (Tanoue et al., 2016). Given that r*Ts*Pmy can suppress the development of acute DSS-induced colitis, it is suggested that r*Ts*Pmy may target the colonic Foxp3^+^ Tregs compartment involved with potential benefits for intestinal homeostasis. Four treatments with r*Ts*Pmy led to the increase of Foxp3^+^ Tregs in the colon, especially in the proximal colon of Foxp3^*eGFP*^ reporter mice (data not shown). However, in the inflammatory condition of colitis, the number of CD4^+^Foxp3^+^ Tregs was reduced in the colon of mice with colitis treated with r*Ts*Pmy compared to those mice with colitis that received PBS only (Figure 5A). Further investigation identified that r*Ts*Pmy actually stimulated thymus-derived Tregs (tTregs) expressed with the transcription factor Helios (Helios^+^) rather than the peripherally derived Tregs (pTregs) in the cLP of mice with DSS-induced colitis (Figure 5B); the former plays a more suppressive and regulatory role in the extreme inflammatory environment (Clough et al., 2020). These results demonstrate that r*Ts*Pmy promotes tTregs expansion but not total Tregs pool in the colon.

To further confirm that r*Ts*Pmy could not induce the *de novo* generation of pTregs under inflammatory conditions, we adoptively transferred CD45RB^*hi*^Foxp3^–^CD4^+^ naïve T cells from Foxp3^*eGFP*^ reporter mice into T/B lymphocyte-deficient Rag1 KO mice to induce T-cell-mediated inflammation in the colon (Chen et al., 2017), followed by treatment with r*Ts*Pmy to assess the differentiation of pTregs in colon in lymphopenia/inflammation. Analysis of pTregs in recipient mice 5 weeks post transfer revealed the sparse population of Foxp3^+^ (eGFP) pTregs converted from transferred naïve T cells in the cLP, mLN (Figure 5C), and spleen (Supplementary Figure 5) in r*Ts*Pmy-treated mice. These results suggest r*Ts*Pmy promotes tTregs expansion but is unable to induce the *de novo* generation of pTregs in the colon.

### r*Ts*Pmy Enhances Tregs Lineage Stability in DSS-Induced Colitis

**FIGURE 6:**
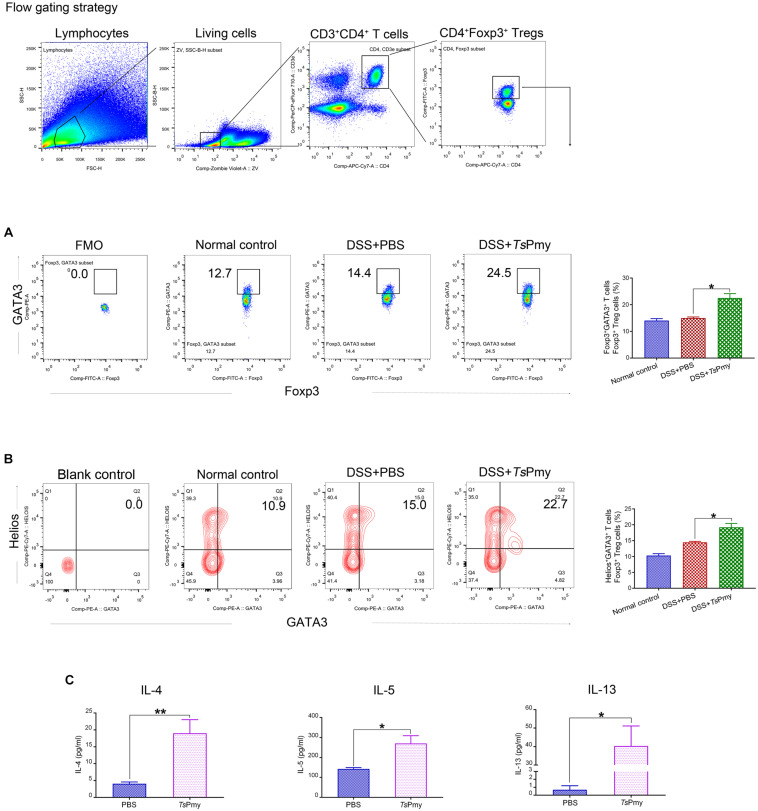
r*Ts*Pmy increases the GATA3^+^Helios^+^ Tregs subset in the Th2 environment. The percentage of GATA3^+^ cells **(A)** and Helios^+^GATA3^+^ cells **(B)** in Foxp3^+^ T cells of cLP from r*Ts*Pmy- or PBS-treated mice. The antibody of GATA3 **(A)** was cut in FMO control samples. Blank control of antibodies of GATA3 and Helios **(B)** was shown. Statistical analyses are shown on the right (*n* = 5). **(C)** The LPMCs were isolated from r*Ts*Pmy- or PBS-treated mice and stimulated with anti-CD3/CD28 mAb for 48 h. IL-4, IL-5, and IL-13 in the culture supernatants were measured by ELISA (*n* = 3). Error bars represent the mean ± SEM. *p < 0.05; **p < 0.01. LPMCs, Lamina propria mononuclear cells. See also in Supplementary Figure 6.

The characteristic Th2 cell transcription factor GATA binding protein 3 (GATA3) forms a complex with Foxp3 to enhance the stability of Tregs and facilitates their accumulation in inflamed intestines (Wohlfert et al., 2011; Rudra et al., 2012). GATA3 expression among Foxp3^+^ Tregs was assessed in this study and results showed that the proportion of Foxp3^+^ Tregs expressing GATA3 was significantly elevated in cLP from r*Ts*Pmy-treated mice with colitis compared with mice with colitis that received only PBS (Figure 6A). Further investigation showed that r*Ts*Pmy increased the GATA3^+^Helios^+^ tTregs subset in cLP of mice with colitis (Figure 6B).

IL-4Rα signaling is crucial for the accumulation of GATA3^+^ Tregs in the inflamed intestine during helminth infections (Abdel et al., 2018); therefore we analyzed the Th2 cytokines IL-4, IL-5, and IL-13 secreted by LPMCs in the colon. LPMCs from r*Ts*Pmy-treated mice secreted higher levels of IL-4, IL-5, and IL-13 (Figure 6C). We also analyzed and found that the proportion of GATA3^+^Foxp3^–^ Th2 subset in the colon was increased in r*Ts*Pmy-treated mice compared with their counterparts (Supplementary Figure 6). Taken together, these results suggest that treatment with r*Ts*Pmy up-regulates GATA3 expression in Foxp3^+^ Tregs in the Th2 environment, possibly enhancing the stability of Tregs in cLP of mice with colitis.

### r*Ts*Pmy Maintains the Suppressive Phenotypes of Tregs in DSS-Induced Colitis

**FIGURE 7:**
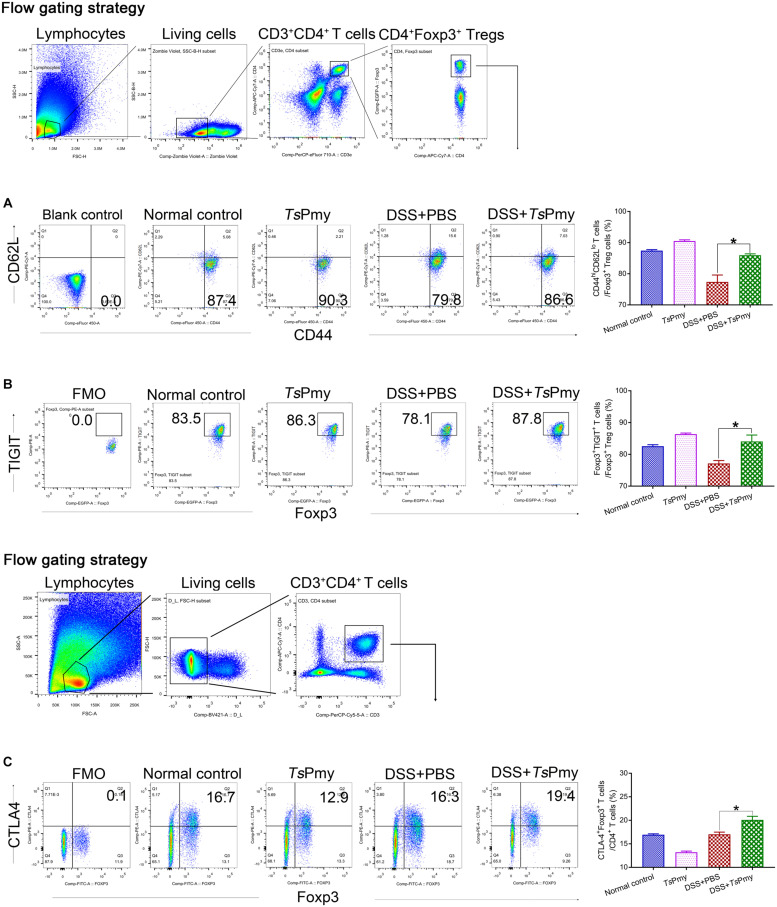
r*Ts*Pmy maintains majority of Tregs in an effective suppressor status (CD62L^*lo*^CD44^*hi*^ eTregs) with greater expression of functional phenotypes of TIGIT and CTLA4 in cLP with DSS-induced colitis. Flow cytometry results showed the frequencies of CD44^*hi*^CD62L^*lo*^ cells **(A)** and TIGIT^+^ cells **(B)** in CD4^+^Foxp3^+^ T cells of cLP from r*Ts*Pmy or PBS-treated mice with or without DSS colitis. The blank control of antibodies of CD62L and CD44 **(A)** was shown, and the antibody of TIGIT **(B)** were cut in FMO control samples. The corresponding percentages are shown on the right (*n* = 5). **(C)** The frequency of Foxp3^+^CTLA4^+^ cells in CD3^+^CD4^+^ T cells. The antibody of CTLA4 was cut in FMO control samples. The corresponding percentage is shown on the right (*n* = 3–5). The bars represent the mean ± SEM. *p < 0.05. cLP, colonic lamina propria.

The Tregs (CD4^+^Foxp3^+^) expressing CD62L^*lo*^CD44^*hi*^ are considered effector regulatory T cells (eTregs) with suppressive functions in inflammatory sites (Tanoue et al., 2016). To determine if r*Ts*Pmy plays a role in maintaining Tregs’ suppressive status in the inflamed colon, the Tregs expressing CD62L^*lo*^CD44^*hi*^ in cLP were measured with flow cytometry. The results showed that the eTregs were decreased in cLP when mice had inflammatory colitis. Treatment with r*Ts*Pmy significantly increased the percentage of eTregs expressing CD62L^*lo*^CD44^*hi*^ in cLP in mice with colitis compared to mice with colitis that received PBS only (Figure 7A), suggesting that r*Ts*Pmy maintains the Tregs in an activated state in the colon. Tregs exert robust suppressive functions via signature phenotype molecules, such as CD25, TIGIT (T-cell immunoreceptor with Ig and ITIM domains), CTLA4 (cytotoxic *T*-lymphocyte antigen 4), and GITR (TNFR-related protein) (Clough et al., 2020). In this study, we found that treatment with r*Ts*Pmy significantly increased the expression of TIGIT (Figure 7B) within CD4^+^Foxp3^+^ T cells and the proportion of CTLA4^+^Foxp3^+^ Treg cells in CD4^+^ T cells (Figure 7C) in mice with colitis compared with mice with colitis that received PBS only.

### Adoptive Transfer of r*Ts*Pmy-Induced Tregs Reduces the Pathology and Inflammation of Colitis in Rag1 KO Mice

**FIGURE 8:**
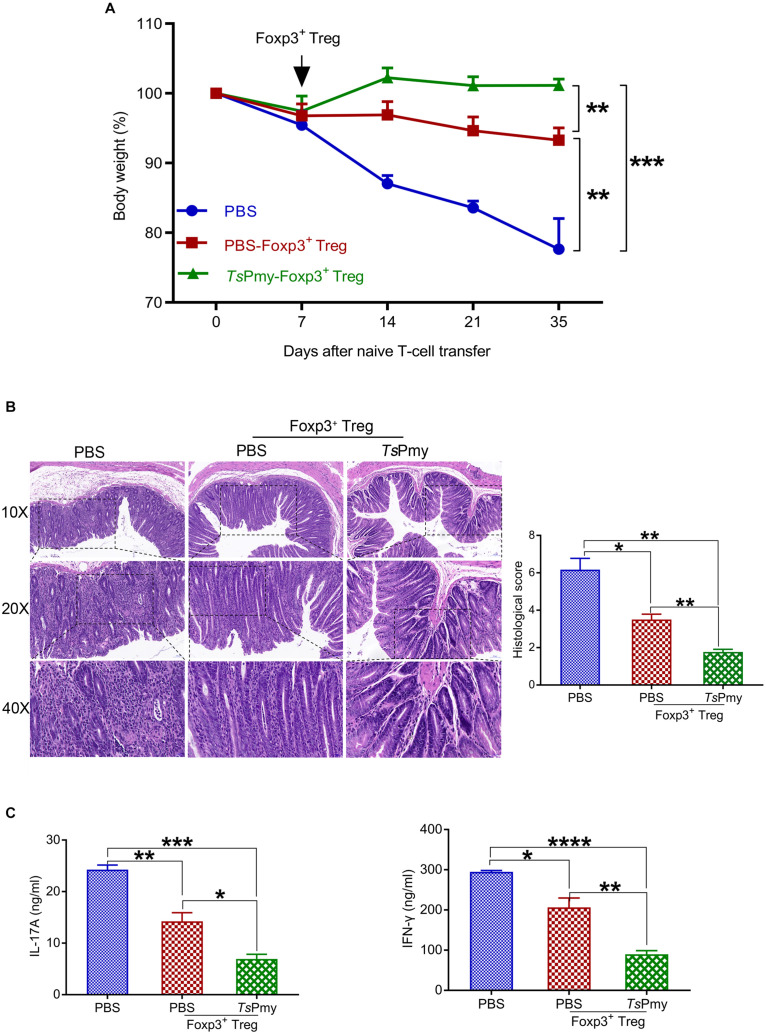
Adoptive transfer of r*Ts*Pmy-induced Tregs reduces the pathology and inflammation of naïve T-cell-induced colitis in Rag1 KO mice. **(A)** Changes in body weight (*n* = 3). **(B)** Hematoxylin and eosin (H&E) images of the distal colon at objective 10x, 20x, and 40x at 4 weeks after adoptive transfer of r*Ts*Pmy- or PBS-treated cLP Tregs compared to Rag1 KO mice receiving PBS. Histological scores of each group are displayed on the right (*n* = 3). **(C)** IL-17A and IFN-γ production of LPMCs in recipient Rag1 KO mice stimulated with anti-CD3/CD28 mAb for 48 h was determined by ELISA (*n* = 3). Data are presented as the mean ± SEM. *p < 0.05; **p < 0.01; ***p < 0.001; ****p < 0.0001. LPMCs, Lamina propria mononuclear cells.

To determine if the inhibitory ability of r*Ts*Pmy-induced Tregs in colitis can be transferred to mice not receiving r*Ts*Pmy, we used a well-defined adoptive transfer model of Rag1 KO mice passively receiving Tregs isolated from cLP of Foxp3^*eGFP*^ reporter mice treated with r*Ts*Pmy or PBS. Chronic colitis was induced in Rag1 KO mice by passive transfer with CD45RB^*hi*^CD4^+^CD25^–^ naïve T cells from C57BL/6J mice exhibiting severe body weight loss (Figure 8A), loss of crypts in the colonic mucosa, infiltration of inflammatory cells (Figure 8B), and higher levels of IL-17A and IFN-γ production by LPMCs (Figure 8C). However, after being adoptively transferred with Tregs from cLP of Foxp3^*eGFP*^ reporter donor mice treated with r*Ts*Pmy or PBS, these pathological conditions were significantly reduced in Rag1 KO mice with T-cell-induced colitis. More strikingly, the reduced pathology was more significant in Rag1 KO mice receiving r*Ts*Pmy-treated Tregs than in those receiving PBS-treated Tregs (Figures 8A–C). The results support that the inhibitory ability of r*Ts*Pmy-induced Tregs is transferable to mice to mitigate the inflammation in colon tissue.

## Discussion

Inflammatory bowel disease is intestinal immune disorder caused by environmental factors and genetic susceptibility. Excessive immune response dominated by Th17 and Th1 is the major driver of intestinal tissue inflammation and damage in IBD patients (Xavier and Podolsky, 2007). Tregs play a critical role in suppressing these immune responses and maintaining immune homeostasis in the intestine (Tanoue et al., 2016). Decrease in Treg number and impaired Treg function have been related to the occurrence of IBD and other inflammatory diseases (Raffin et al., 2020). Immunomodulatory effects of helminth infection and helminth-derived molecules have been widely applied in the experimental immunotherapy of allergic or autoimmune inflammatory diseases; some of them have even been used in the clinical trials of allergic diseases (Feary et al., 2010; Daveson et al., 2011) or IBD (Summers et al., 2005; Mortimer et al., 2006). “Helminth therapy” in IBD includes the use of viable ova/larvae, helminth-derived crude extracts, purified molecules and cellular immunotherapy with helminth extract/antigen-pulsed immune cells (Lopes et al., 2016).

In this study, we demonstrated that treatment with r*Ts*Pmy, the major immunomodulatory protein expressed by *T. spiralis*, significantly reduced the development of DSS-induced colitis in a mouse model, including improvements in clinical manifestations and mitigation of pathology in the inflamed colon (Figures 1, 2). We observed the dynamic changes in CD4^+^ effector T cells involved in the pathological process of DSS-induced colitis in the mouse colon. Upon exposure to DSS, the effector CD4^+^ T cells are recruited to the ILFs in the colon. As disease progresses, the influx of CD4^+^ effector T cells from ILFs to the colonic mucosa leads to mucosal erosion (Figure 3A). After being treated with r*Ts*Pmy, the recruitment of the CD4^+^ effector T cells was significantly reduced and the pathological process of DSS-induced colitis was mitigated (Figure 3B). The activated excessive CD4^+^ effector T cells producing IL-17A and IFN-γ (Th17 and Th1) are believed to play pivotal roles in the pathogenesis of IBD (Xavier and Podolsky, 2007). The reduced pathology is correlated with reductions in Th1 and Th17 cells, neutrophil infiltration, and the corresponding cytokines such as IL-17A, IL-17F, and IL-6 in colonic LPMCs (Figures 2, 4). Even though we found that r*Ts*Pmy reduced the proportion of Th1 (CD4^+^IFN-γ^+^) cells in LPMCs, the IFN-γ level secreted by LPMCs was increased regardless of reductions in other pro-inflammatory cytokines IL-17A, IL-17F, and IL-6. The increased level of IFN-γ in colitis is possibly related to the increased level of T-bet that we observed in r*Ts*Pmy-treated colonic Tregs during colitis (data not shown). Tregs have been shown to up-regulate the Th1-related transcription factor T-bet among Tregs that may stimulate the secretion of the pro-inflammatory cytokine IFN-γ (Di Giovangiulio et al., 2019). Interestingly, we observed significantly increased IL-10 and TGF-β levels secreted by LPMCs of mice with colitis treated with r*Ts*Pmy, indicating the regulatory pathway may be involved in the reduced Th1/Th17 inflammatory response induced by the treatment of r*Ts*Pmy (Figure 4), which is consistent with the previous study that recombinant *Schistosoma japonicum* secreted protein Sj16 diminishes pro-inflammatory cytokine production but up-regulates immunoregulatory cytokine production IL-10 and TGF-β (Wang et al., 2017) in colonic mucosa. Therefore, we would like to explore if the regulatory T cells (Tregs) are involved in the alleviation of colitis during treatment with r*Ts*Pmy.

Tregs are critical for maintaining intestinal immune homeostasis, especially in the colon (Tanoue et al., 2016). It has been observed that helminth infections elevate the number of colonic Tregs and Foxp3 expression (Taylor et al., 2012) that are important in maintaining the immune balance in the colon (Gause and Maizels, 2016). In our study, we observed significantly increased abundance of Tregs in the colon in Foxp3^*eGFP*^ reporter mice after treatment with r*Ts*Pmy (data not shown), and these Tregs were able to significantly inhibit chronic colitis induced by naïve T cells in Rag1 KO mice (Figure 8). However, the number of total Tregs was not increased in the colon of mice with colitis treated with r*Ts*Pmy (Figure 5A) even though the levels of IL-10 and TGF-β were significantly higher in the inflamed colon (Figure 4C). Further investigation of the phenotype of Tregs found that the thymus-derived Tregs (tTregs) expressed with Helios were significantly increased in cLP of mice with DSS-induced colitis treated with r*Ts*Pmy (Figure 5B). Passive transfer with naïve T cells from Foxp3^*eGFP*^ reporter mice into T/B lymphocyte cell-deficient Rag1 KO mice followed by the treatment of r*Ts*Pmy showed that treatment with r*Ts*Pmy did not induce, and even inhibited, the *de novo* generation of pTregs in the colon of recipient mice (Figure 5C). The results indicate that r*Ts*Pmy only promotes tTregs expansion but not the differentiation of pTregs in the colon in an inflammatory environment. Colonic Foxp3^+^ Tregs comprise thymus-derived Tregs (tTregs) and peripherally derived Tregs (pTregs) (Tanoue et al., 2016). The tTregs complete their development in the thymus where they escape negative selection and then migrate to the colon, while pTregs are generated from naïve T cells in the colon microenvironment that is rich in TGF-β, microbial antigens and metabolites (Tanoue et al., 2016). The tTregs, rather than pTregs maintain stable suppressive activity in the extreme inflammatory environment (Clough et al., 2020). The specific stimulation of tTregs by r*Ts*Pmy indicates the specific regulation of tTregs in the inflamed colon by helminth-derived protein. A recent study reported that *Nippostrongylus brasiliensis* and *Heligmosomoides polygyrus bakeri* drove the ST2^+^GATA3^+^ tTreg responses in intestinal mucosa through DC-derived IL-33 that suppressed helminth immunity (Schiering et al., 2014; Hung et al., 2020). During helminth infection, tTregs are believed to produce the initial regulation of Th2 protective immune responses, while pTregs are developed more slowly. Mice depleted of natural Tregs prior to filarial or *S. mansoni* infections showed an increased Th2-mediated worm expulsion, suggesting that tTregs play an important role in the early stage of infection (Hesse et al., 2004; Baumgart et al., 2006). More studies showed that helminth infection rapidly expanded natural CD4^+^Foxp3^+^ regulatory T cells (tTregs) to inhibit host protective immunity against helminth infection while adaptive pTregs were differentiated slowly with limited protection against helminth infection (Taylor et al., 2012). The adaptive pTregs responded slowly to helminth infection through a TGF-β mimic derived from a helminth antigen and may suppress protective immunity during a later infection stage (Grainger et al., 2010). Blockade of TGF-βR signaling after 4 weeks of *H. polygyrus* infection inhibited the generation of pTregs thereby increasing parasite killing (Grainger et al., 2010). The tTregs and pTregs may have distinct yet overlapping functions as regulators during helminth infection. In this study, we demonstrated that r*Ts*Pmy induced and expanded tTregs in the colon; however, it is still unknown if the induced tTregs are r*Ts*Pmy antigen-specific. It will be important to determine the antigenic specificity of r*Ts*Pmy-activated tTregs and its specific role in the inhibition of inflammatory diseases.

After we showed that r*Ts*Pmy was able to stimulate and expand the tTregs in colitis, the question remains if r*Ts*Pmy can maintain the stability of Tregs in the colon so they can exert their regulatory functions. The tTregs expressing GATA3 exhibit stable suppressive activity and do not undergo reprogramming in the extreme inflammatory environment (Tanoue et al., 2016). Intrinsic GATA3 expression by Tregs is essential to sustain high levels of Foxp3 expression in an inflammatory setting and is required for efficient accumulation of Tregs at inflamed sites (Rudra et al., 2012). In this study, we showed that treatment with r*Ts*Pmy significantly increased GATA3 expression in Foxp3^+^ Tregs in cLP of mice with colitis (Figure 6A). Nearly all GATA3^+^ Tregs also expressed Helios in this study (Figure 6B), indicating their origin in tTregs. These r*Ts*Pmy-induced GATA3^+^Helios^+^ Tregs should be a functional tTregs subset with more stability (Tanoue et al., 2016). It is believed that IL-4Rα signaling is crucial to up-regulate GATA3 in Tregs and promote the accumulation of GATA3^+^ Tregs in the inflamed intestine during helminth infection (Abdel et al., 2018). We investigated Th2 responses in LPMCs and found that r*Ts*Pmy elicited robust Th2 immune responses in the colon including strong secretion of IL-4, IL-5, and IL-13 (Figure 6C). The results indicate that the r*Ts*Pmy-induced Th2 environment facilitates GATA3 expression in Tregs, and therefore increases the stability of Tregs in the colon to exert a suppressive function on DSS-induced colitis.

To determine if r*Ts*Pmy plays a role in stimulating Tregs to maintain their suppressive status in inflammatory colon, we measured the effector regulatory T cells (eTregs) which express CD62L^*lo*^CD44^*hi*^ (Tanoue et al., 2016) in the colon in colitis. Our results identified that r*Ts*Pmy significantly increased the percentage of eTregs expressing CD62L^*lo*^CD44^*hi*^ in cLP of mice with colitis compared to mice treated with PBS only. The increased eTregs also expressed suppressive signature phenotype molecules of TIGIT and CTLA4 (Joller et al., 2014; Clough et al., 2020; Figure 7), further suggesting r*Ts*Pmy up-regulates eTregs to maintain effective suppressive status in the inflammatory colon. However, the increased expression of CTLA4 was observed only in CD4^+^Foxp3^+^ T cells in colitis. In normal mice, treatment with r*Ts*Pmy increased only CTLA4 in CD4^+^Foxp3^–^ T cells (data not shown), not in CD4^+^Foxp3^+^ T cells, which is consistent with the results of infections with *H. polygyrus*, *Strongyloides ratti*, *Brugia malayi*, and *Litomosoides sigmodontis* (Finney et al., 2007; Taylor et al., 2007; Walsh et al., 2007; McSorley et al., 2008). The up-regulation of CTLA4 in Tregs in inflammatory conditions indicates that the helminth-secreted immunomodulatory molecules regulate Treg function more effectively when there is a greater degree of inflammation (D’Elia et al., 2009).

The suppressive effect of r*Ts*Pmy-induced Tregs was also confirmed by passive transfer to Rag1 KO mice with chronic colitis induced by CD45RB^*hi*^CD4^+^CD25^–^ naïve T cells; they had fewer clinical manifestations and pathological damage in colon that was associated with reduced levels of IL-17A and IFN-γ production in colonic LPMCs. The findings further confirm that the therapeutic efficacy of r*Ts*Pmy in colitis results from induction of Tregs. The results are consistent with the investigation of *H. polygyrus* infection-induced Tregs, in which adoptive transfer of Tregs from the cLP of *H. polygyrus*-infected mice as opposed to uninfected-mice significantly inhibited chronic colitis induced by CD4^+^CD25^–^ T cells in Rag1 KO mice (Hang et al., 2013).

## Conclusion

We demonstrated the prophylactic and therapeutic effects of r*Ts*Pmy in the experimental colitis associated with colonic Tregs activation in this study. The therapeutic effect of r*Ts*Pmy-induced Tregs was transferrable to mice with chronic colitis. The more significant finding in this study is that r*Ts*Pmy specifically stimulated the differentiation and expansion of thymic-derived Tregs but not peripherally derived Tregs; the former play more suppressive and regulatory roles in the extreme inflammatory environment. Treatment with r*Ts*Pmy also enhanced the differentiation of effector Tregs with greater suppressive function in the colon with colitis. In addition, r*Ts*Pmy enhanced the stability of effective Tregs in the inflamed colon by stimulating the expression of GATA3 on Foxp3^+^ Tregs in Th2 environment. The findings in this study describe the systemic and dynamic activation of effective Tregs induced by a major immunomodulatory protein r*Ts*Pmy and the roles of r*Ts*Pmy-induced Tregs in maintaining gut immune homeostasis during inflammation, providing further insight into the immunological mechanisms involved in the therapeutic effect of helminth-derived proteins in inflammatory bowel diseases.

## Data Availability Statement

The original contributions presented in the study are included in the article/Supplementary Material, further inquiries can be directed to the corresponding author/s.

## Ethics Statement

The animal study was reviewed and approved by the Capital Medical University Animal Care and Use Committee under projects AEEI-2017-140, AEEI-2016-008, and AEEI-2017-133.

## Author Contributions

CH: investigation, methodology, formal analysis, and writing – original draft. WW: validation and methodology. BZ: writing – review and editing. ZW: methodology. JH: resources. XS and XZ: conceptualization, supervision, funding acquisition, and writing – review and editing. All authors discussed the results, commented on the manuscript text, and approved the final version submitted.

## Conflict of Interest

The authors declare that the research was conducted in the absence of any commercial or financial relationships that could be construed as a potential conflict of interest.
