# Supplementary material for: Trichinella spiralis Paramyosin Induces Colonic Regulatory T Cells to Mitigate Inflammatory Bowel Disease
Source: Front Cell Dev Biol. 2021 Jul 15;9:695015. doi: 10.3389/fcell.2021.695015 (PMC8320175; doi:10.3389/fcell.2021.695015)
Supplement: Supplementary file 1 [file Data_Sheet_1.docx]

Figures and Figures legend


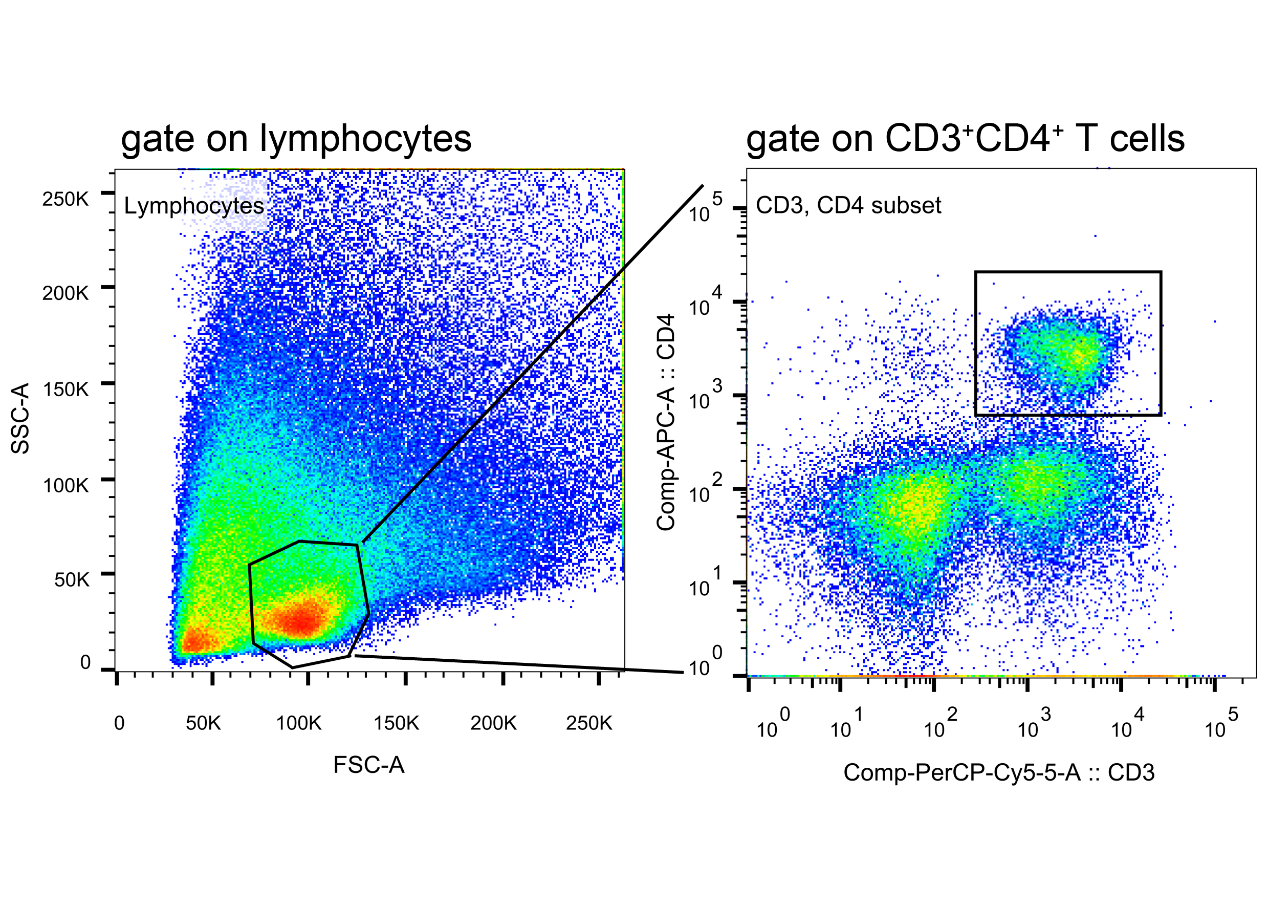


Figure S1, related to Figure 4A-B. The gate strategy of Th17 and Th1 cells among CD3^+^CD4^+^ T cells in colonic lamina propria for flow cytometry.


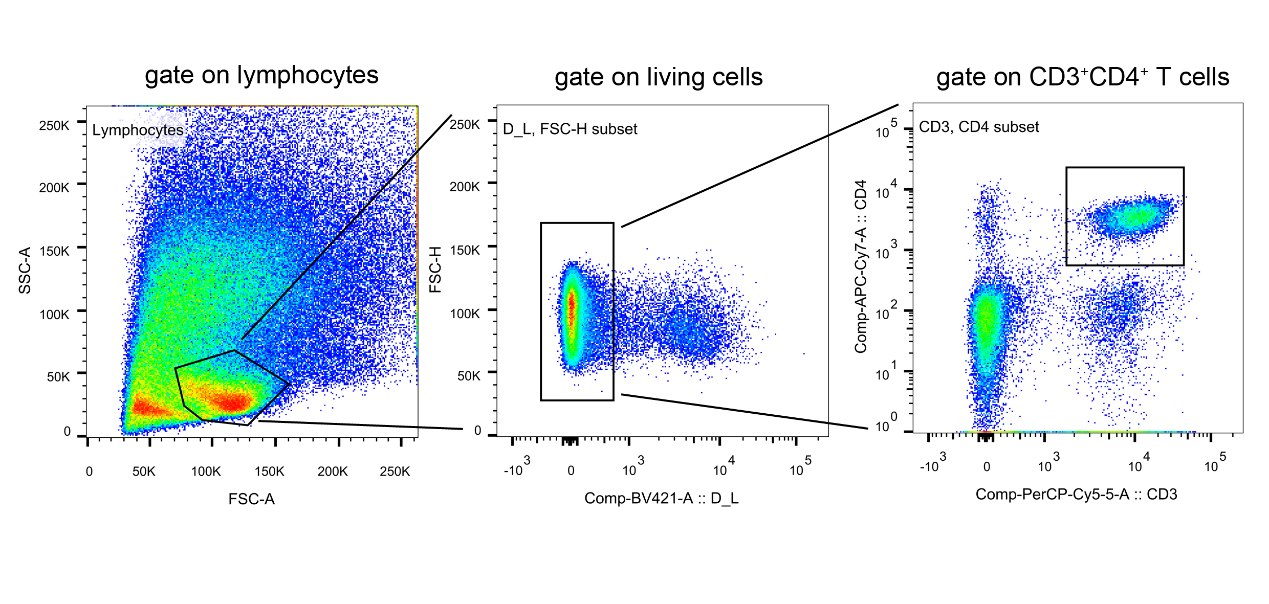


Figure S2, related to Figure 5A. The gate strategy of Treg cells among CD3^+^CD4^+^ T cells in colonic lamina propria for flow cytometry.


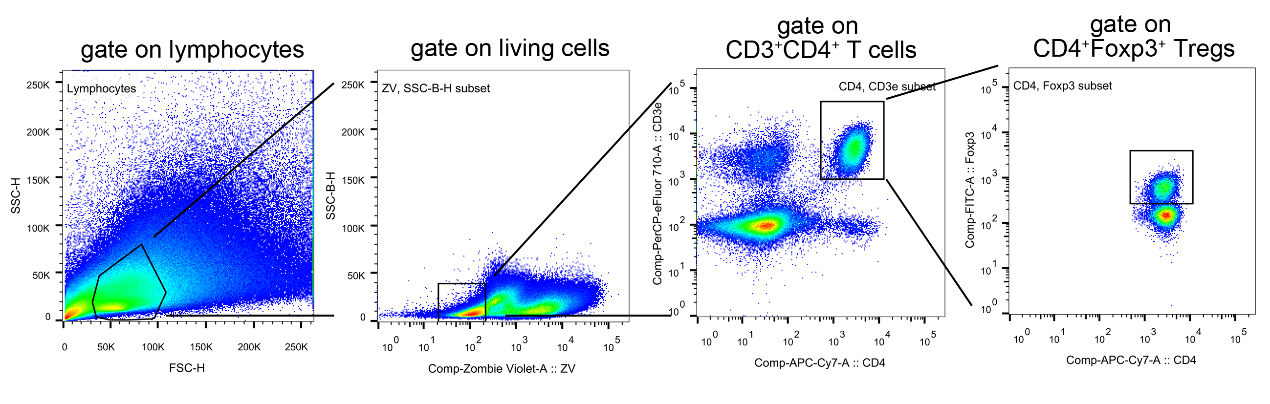


Figure S3, related to Figure 5B, Figure 6A-B. The gate strategy of Helios^+^, GATA3^+^, and Helios^+^GATA3^+^ Treg cells among total Treg pool in colonic lamina propria for flow cytometry.


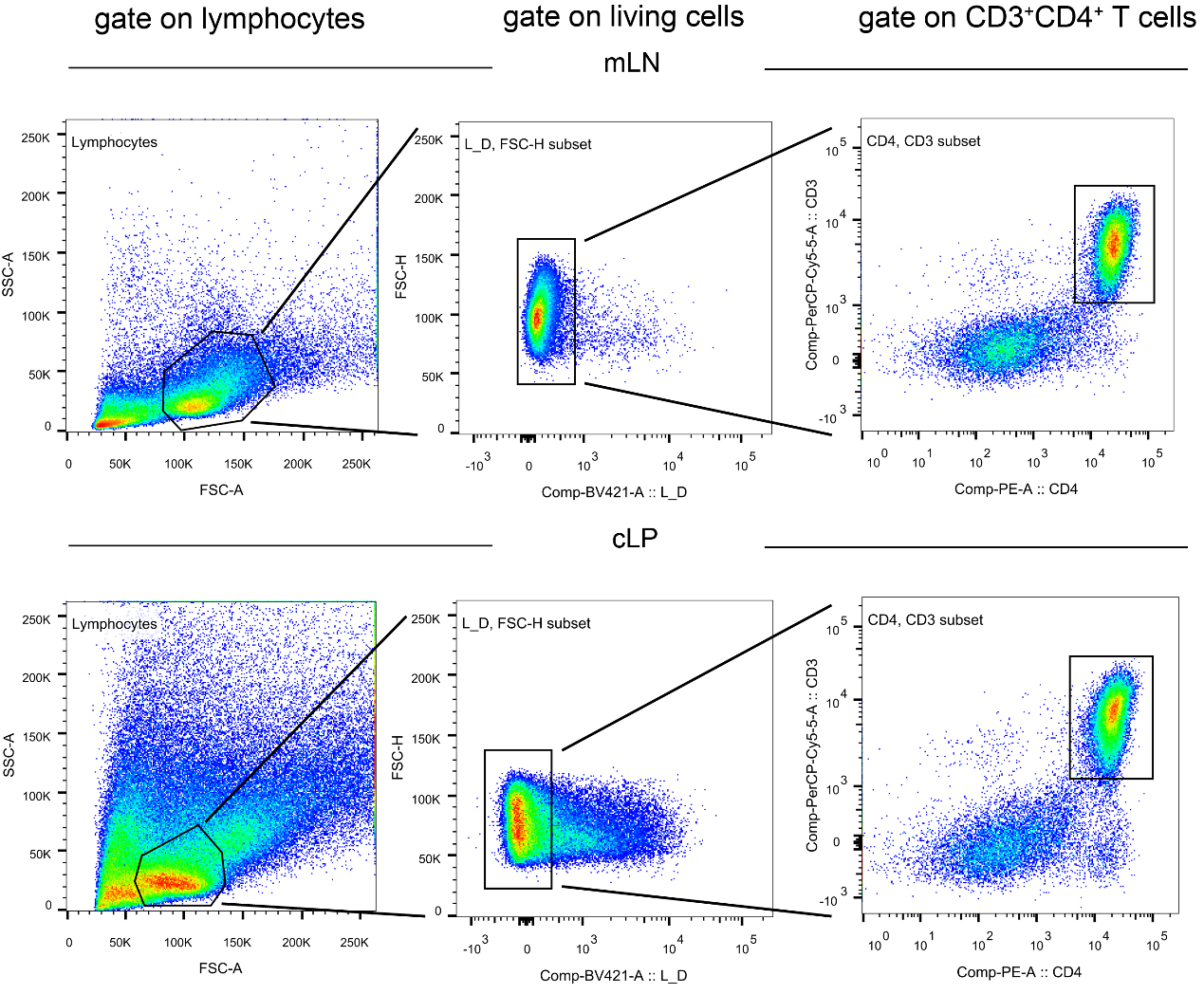


Figure S4, related to Figure 5C. The gate strategy of Treg cells for flow cytometry in mLN and cLP of Rag1 KO mice. mLN, mesenteric lymph nodes. cLP, colonic lamina propria


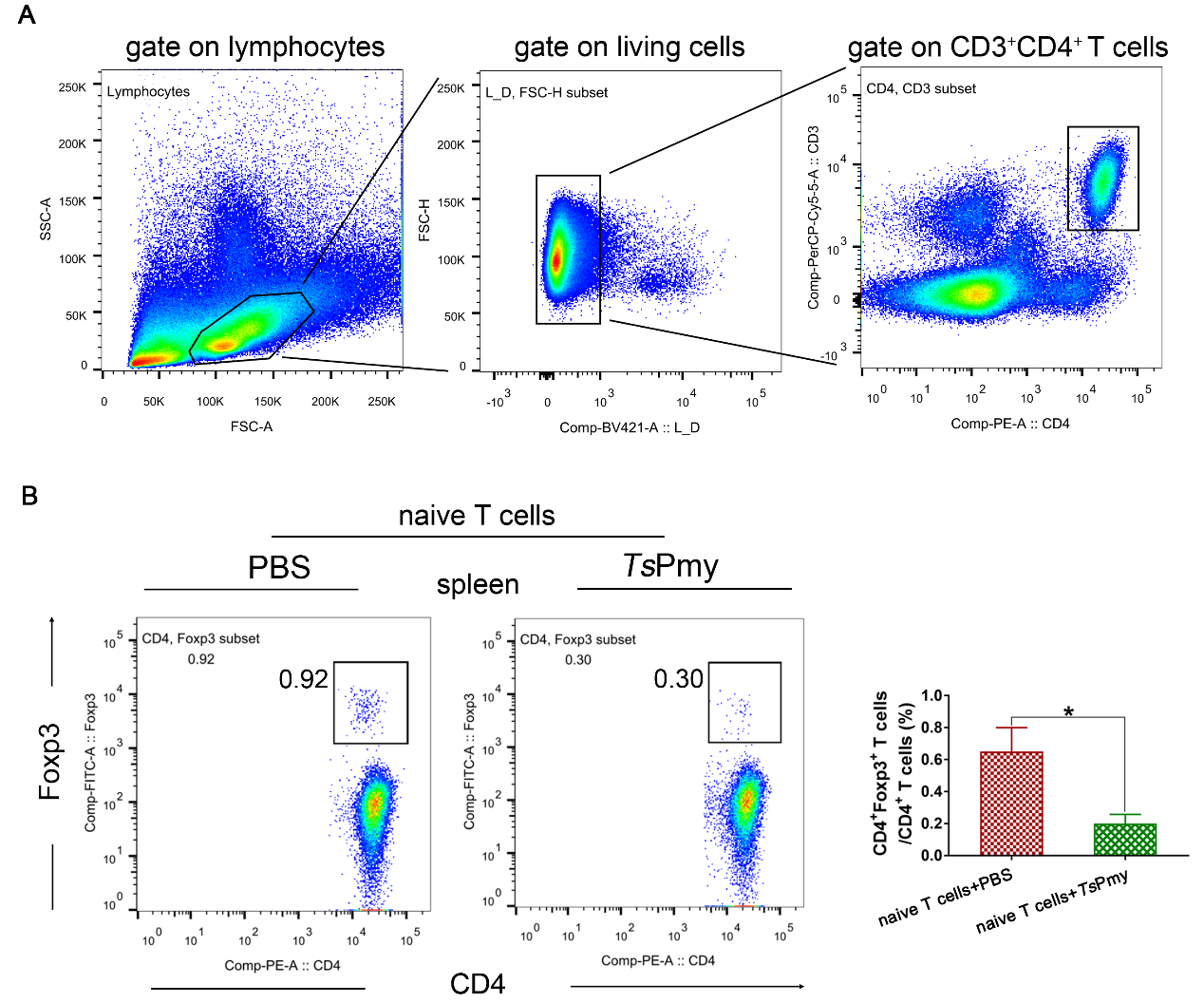


Figure S5, related to Figure 5C. *Ts*Pmy could not induce pTreg differentiation in spleen with Rag1 KO chronic colitis. **(A)** The gate strategy for flow cytometry. **(B)** The percentages of Foxp3^+^ (eGFP) T cells in CD3^+^CD4^+^ T cells of spleen at 5 weeks after adoptive transfer of naïve T cells into Rag1 KO mice. Statistical analysis is shown on the right (n = 3). Error bars represent the mean ± SEM. *p < 0.05.


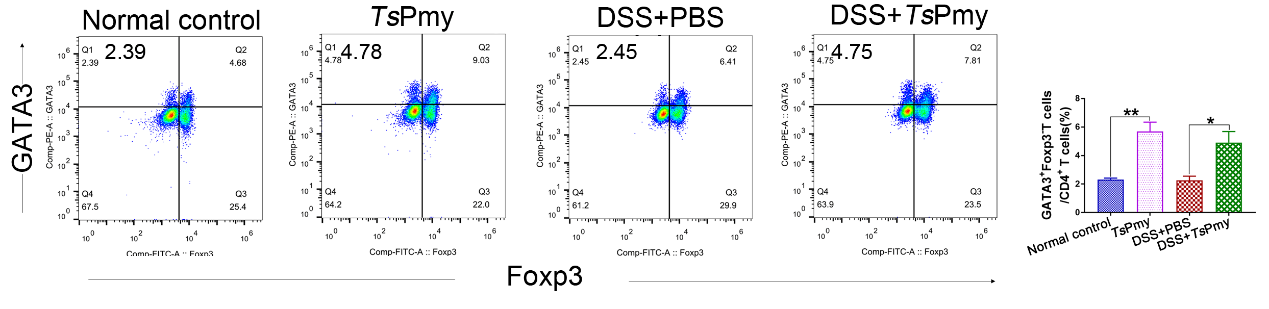


Figure S6, related to Figure 6C. *Ts*Pmy increases the proportion of GATA3^+^Foxp3^-^ Th2 cells of cLP with or without DSS colitis. The gate strategy for flow cytometry is shown in Figure S8. Statistical analysis of the percentages of GATA3^+^Foxp3^-^ Th2 cells among CD3^+^CD4^+^ T cells is shown on the right (n = 5). Error bars represent the mean ± SEM. *p < 0.05; **p < 0.01. cLP, colonic lamina propria.
